# Infrared avalanche photodiodes from bulk to 2D materials

**DOI:** 10.1038/s41377-023-01259-3

**Published:** 2023-08-31

**Authors:** Piotr Martyniuk, Peng Wang, Antoni Rogalski, Yue Gu, Ruiqi Jiang, Fang Wang, Weida Hu

**Affiliations:** 1 Institute of Applied Physics, Military University of Technology, 2 Kaliskiego Street, 00-908, Warsaw, Poland; 2 State Key Laboratory of Infrared Physics, Shanghai Institute of Technical Physics, Chinese Academy of Sciences, 500 Yu-Tian Road, Shanghai, China

**Keywords:** Optoelectronic devices and components, Optical materials and structures

## Abstract

Avalanche photodiodes (APDs) have drawn huge interest in recent years and have been extensively used in a range of fields including the most important one—optical communication systems due to their time responses and high sensitivities. This article shows the evolution and the recent development of A^III^B^V^, A^II^B^VI^, and potential alternatives to formerly mentioned—“*third wave*” superlattices (SL) and two-dimensional (2D) materials infrared (IR) APDs. In the beginning, the APDs fundamental operating principle is demonstrated together with progress in architecture. It is shown that the APDs evolution has moved the device’s performance towards higher bandwidths, lower noise, and higher gain-bandwidth products. The material properties to reach both high gain and low excess noise for devices operating in different wavelength ranges were also considered showing the future progress and the research direction. More attention was paid to advances in A^III^B^V^ APDs, such as AlInAsSb, which may be used in future optical communications, type-II superlattice (T2SLs, “Ga-based” and “Ga-free”), and 2D materials-based IR APDs. The latter—atomically thin 2D materials exhibit huge potential in APDs and could be considered as an alternative material to the well-known, sophisticated, and developed A^III^B^V^ APD technologies to include single-photon detection mode. That is related to the fact that conventional bulk materials APDs’ performance is restricted by reasonably high dark currents. One approach to resolve that problem seems to be implementing low-dimensional materials and structures as the APDs’ active regions. The Schottky barrier and atomic level thicknesses lead to the 2D APD dark current significant suppression. What is more, APDs can operate within visible (VIS), near-infrared (NIR)/mid-wavelength infrared range (MWIR), with a responsivity ~80 A/W, external quantum efficiency ~24.8%, gain ~10^5^ for MWIR [wavelength, *λ* = 4 μm, temperature, *T* = 10–180 K, Black Phosphorous (BP)/InSe APD]. It is believed that the 2D APD could prove themselves to be an alternative providing a viable method for device fabrication with simultaneous high-performance—sensitivity and low excess noise.

## Introduction

The avalanche multiplication effect can be used to detect low-power optical signals and even single-photons due to the amplification mechanism within all main: near- (NIR), short- (SWIR), mid- (MWIR), and long wavelength infrared radiation (LWIR) ranges. An advanced laser radar and weapons systems implemented in long-range army and space applications must detect, recognize and track various targets under a diversity of atmospheric conditions including absorption by CO, CO_2_, and H_2_O vapor, which leads to significant signal attenuation in the optical system. That output signal suppression requires an extra amplifier along with a system to correctly detect the signal at the detector stage. The devices based on avalanche photodiodes (APDs) exhibiting high bandwidth (*BW*) and gain (*M*)—high gain-bandwidth product (*GBW*) and low excess noise [*F(M)*] at the same time are well matched to detect suppressed optical signals, e.g., in the long-distance applications such as free-space optical communications (FSO), night vision, light detection, and ranging (LIDAR/LADAR), time of flight (ToF), intelligent robotic and finally in battlefield conditions (military applications). Therefore, improvement in *GBW* and *F(M)* reduction has been a key goal for APD’s progress. The methods to suppress the *F(M)* may be divided into three tactics. The initial approach could be to choose a material (to include “*third wave*” materials and their technologies) with advantageous multiplication properties. Next, the *F(M)* may be substantially limited by the reduction of the avalanche layer to use the non-local effect of the multiplication phenomena. The final method may be widely categorized as impact ionization engineering (*I*_*2*_*E*) exploiting properly constructed heterojunctions.

The APD materials’ selection is conditioned by the potential applications to include the most common: high-speed receivers, single-photon counters, and laser range finders^1,2^. In the field of fiber optic communication (FOC), InGaAs ternary alloy is much more expensive in terms of fabrication than Ge, but provides lower noise and higher time response. The Ge APDs are advised for detection systems where noise generated by the amplifier is high. The development of device technology with active regions built of the narrow gap semiconductors, such as HgCdTe and T2SLs (“*third wave*” material/technology) has contributed to the development of the new passive/active detection applications and capabilities. In active imagery systems, a laser source is implemented to the observed region, and reflected radiation is temporally examined. The output signal may be amplified in the APD itself, before going to the readout integrated circuit (ROIC). In addition, the grouping of dual-band capability and multiplication gain is another technology enabling dual-band detection for a wide temperature selection.

**Fig. 1:**
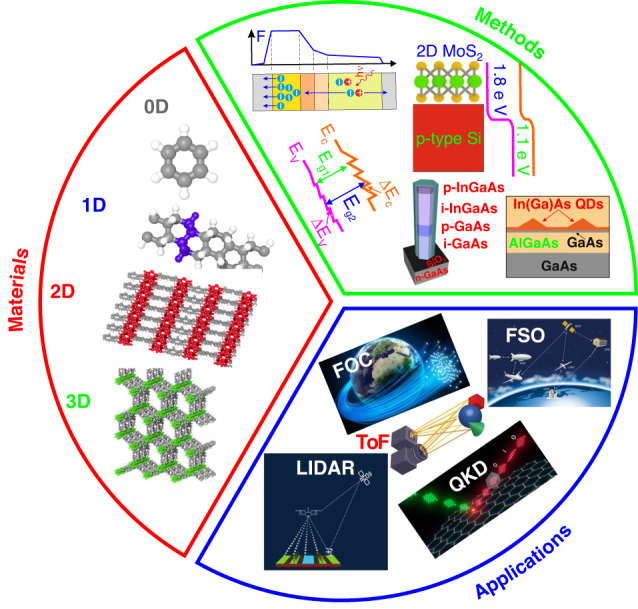
Bulk to low-dimensional material, tactics to fabricate APDs and possible applications: FOC, FSO, LIDAR and QKDs. Methods, technologies, and applications roadmap for avalanche photon-sensing technologies starting from bulk to low-dimensional materials

The APD could operate under the conditions where applied bias is higher than the infinite gain voltage meaning that the appearance of the single-photon causes an avalanche breakdown producing a high signal marking the presence of another photon (passive or active imaging). This mode of operation is referred to as a counting or single-photon avalanche detector (SPAD)—called a Geiger mode avalanche detector by Cova et al. pioneering paper^3^. The SPAD is more sensitive than a photomultiplier, however, when the avalanche process is initiated at infinite gain, additional photons detected during the pulse and circuit regeneration are discounted which makes SPAD more like a Geiger counter than a photomultiplier. SPADs build a variety of approaches to reach single-photon detection (SPD) mode and compete with superconducting nanowire single-photon detectors (SNSPDs). The main reason for this tendency is unquestionably the move to optical quantum information applications—quantum key distribution (QKD) putting severe requirements on detector parameters that move away from the performance of the well-developed typical APDs. Effective single-photon numeration, with a single-photon detection efficiency (SPDE) > 50%, was reached just for wavelengths shorter than <2 μm^4^. SNSPDs exhibit outstanding performance on a wide wavelength range, but their applications is restricted by cryogenic cooling requirements. In contrast, SPADs circumvent the SNSPDs’ fundamental restrictions by offering a reasonable option at ≤300 K mainly by A^III^B^V^ material leader—InGaAs. Reaching the high performance in the MWIR exhibits potential in astronomy, LIDAR, dark matter research applications, and examination of chemistry and molecular dynamics, to include many absorption fingerprints for molecules: H_2_O, CO_2_, O_2_, O_3_, CH_4,_ and N_2_O_3_^5^. Figure 1 illustrates the significance of these devices pointing to the technology roadmap development from typical bulk to low-dimensional APDs to include SPDs^6–8^.

The focal plane array (FPA) intended to operate in LWIR is advantageous because the number of photons in the 8–12 μm atmospheric transparency window is significant for reaching high detectivity and response time. In addition, astronomy applications need FPAs exhibiting high *M* and low *F(M)*, to detect low radiation flux from far located stars. The avalanche ionization in LWIR can be more simply reached in comparison with SWIR and MWIR devices. Even though a higher *M* may be reached under a given bias, the large dark current is an issue for LWIR APDs significantly impacting the device performance. Derelle et al. presented the *n*^*+*^*/n*^−^*/p* planar APD deposited by molecular beam epitaxy (MBE), exhibiting *M* = 16 at −2.7 V and cut-off wavelength, *λ*_*c*_ ~ 9 μm at 80 K. Authors showed that the *F(M)* assessment in HgCdTe LWIR APDs is restricted to low *M* [*F(M)* = 1–1.25 at *M* = 6] caused by tunneling currents influence^9^. Since the photocurrent to dark current ratio is low at high reverse voltage in LWIR it is difficult to estimate *F(M)* with high *M*^10,11^.

The member of the “*third wave*” group—two-dimensional (2D) layered materials and van der Waals (vdW) heterostructures can be also used to fabricate avalanche multiplication to include single-photon-counting technologies. Recently, the spectacular growth in the quantity of research papers related to the promising 2D photodetectors has been observed, however, those materials exhibit low absorption caused by their thin atomistical nature. To use those unique 2D materials properties for device design, the considerable latest attempts have been directed at combining with photonic structures (dielectric waveguides), plasmonic structures, or photonic crystals. That combination with photonic structures allowed to demonstrate single graphene layer with high absorption, modulators, detectors, and lasers^12^. Impact ionization leading to the carrier avalanche is a favorable approach to fabricating 2D photodetectors exhibiting high detection efficiency.

In comparison to standard bulk, the 2D materials exhibit numerous exceptional capabilities, such as mechanical flexibility, strong light-matter coupling, self-passivated surfaces, and gate-tunable Fermi-level providing flexibility in heterostructure design^13,14^. Those materials are characterized by different impact ionization coefficients versus carrier transport direction. The electric field needed for avalanche multiplication in out-of-plane transport is hundreds of kV/cm, while for in-plane close to tens of kV/cm is confirmed by measured results^15^. The 2D layered gapless graphene can detect radiation from ultraviolet (UV) to microwave making it an alternative for numerous photodetector designs operating in wide spectral ranges, however, its zero-bandgap characteristics limit the fabrication of photodetectors with high detectivity. Alternatively, 2D transition metal dichalcogenides (TMDs), thickness-dependent energy bandgap MoS_2_ and WSe_2_, exhibit promising photodetection capabilities mainly in the visible (VIS) to NIR ranges to include impact ionization effect. In comparison to the graphene and TMDs, 2D black phosphorus (BP) exhibiting a direct energy bandgap within the range from 0.3 eV (bulk) to 2 eV (monolayer) proved to be a proper material candidate for APD technology^8^. The multiplication was also observed in 2D InSe for the VIS range. In addition, not only the conventional impact ionization effect but also the ballistic avalanche mechanism was observed in the 2D materials family. The effectiveness of the multiplication mechanism varies versus the material’s intrinsic capabilities. Consequently, the research of innovative materials characterized by the low electric field for avalanche multiplication is significant in reaching energy-effective devices. The avalanche multiplication mechanism in conventional materials is restricted by high bias which could be circumvented by 2D materials-based APDs^15^.

Taking the above into consideration this paper shows the current status and future development of IR-based APDs. It encompasses both bulk HgCdTe and A^III^B^V^based material systems including well-known “*third wave*” material family member—superlattices (SLs). In addition, the current progress in the new materials and architectures for high-performance IR APDs is presented to include innovative “*third wave*” 2D materials. In addition, the strategies to reach high-performance APDs are presented. The field related to the APD advances in telecommunications is generally omitted due to the excellent review papers published recently^16,17^.

## Background

**Fig. 2:**
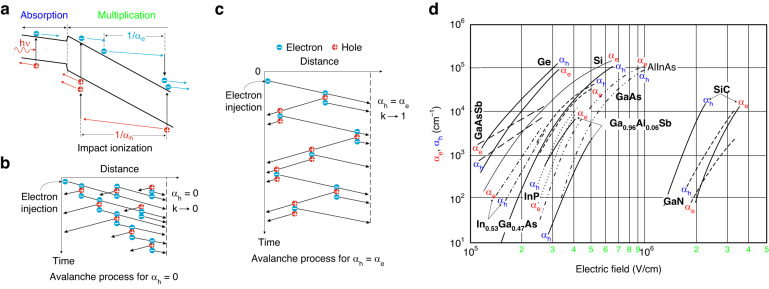
The APD’s operating principle. **a** Electron and hole multiplication mechanisms, schematic of multiplication mechanism for **b** *k* = 0 (*α*_*h*_ = 0) and **c** *k* = 1 (*α*_*e*_ = *α*_*h*_), where *k* = *α*_*h*_/*α*_*e*_ – *α*_*e*_, *α*_*h*_ represent electron and hole multiplication coefficients. **d** *α*_*e*_, *α*_*h*_ ionization coefficients versus electric field for selected semiconductors used for APDs’ fabrication^26^

Figure 2(a–c) presents the diagram of the multiplication effect in APD, where the internal gain is obtained through the avalanche mechanisms generated by the stochastic impact ionization process inherently accompanied by *F(M)* deterioration limiting *GBW*. This is because both carriers (electron and hole) may be multiplicated. The carriers are photogenerated in the low electric field active layer and, ideally, only the carriers exhibiting the utmost impact ionization probability are moved toward the high-electric field multiplication area.

The carrier’s ability to multiply is given by the *α*_*e*_ (electrons) and *α*_*h*_ (holes) ionization coefficients. Those parameters describe the multiplication probability per unit length meaning that ~1/*α*_*e*_ and ~1/*α*_*h*_ represent an average distance carrier moves before impact ionization occurs [see Fig. 2(a)]. The electron and hole ionization coefficients, thus, *k* = *α*_*h*_/*α*_*e*_, are conditioned by the material properties like the carriers’ effective masses and scattering mechanisms. The electron and hole ionization coefficients rise versus voltage and decline versus temperature. The rise in voltage is driven by extra carrier velocity under an electric field, while the decrease versus temperature relates to the non-ionizing collisions with thermally excited atoms. However, there are reports about positive temperature variation of In_0.53_Ga_0.47_As impact ionization^18^. The ionization coefficients follow the Chynoweth model exhibiting exponential dependence on the electric field (for a given temperature):

1 $$\alpha ={aexp}\left(-{\left[\frac{b}{E}\right]}^{c}\right),$$

where: *E* is an electric field in the multiplication area and *a*, *b*, *c* are measured constants.

The carriers’ ionization coefficient ratio, *k* = α_*h*_/α_*e*_ is considered an important parameter characterizing the APD’s performance. When holes do not multiplicate significantly (α_*h*_ « α_*e*_, *k* → 0), the avalanche ionization is driven by electrons. As Fig. 2(b) depicts the avalanche process proceeds from left to right and ends after all electrons reach the n-type part of the depletion layer. When both carriers multiplicate (*k* → 1), the holes are transported to the left creating electrons being moved to the right generating more holes transported to the left, in a feasibly infinite cycle. The impact ionization effect for *k* = 1 is chain-like [see Fig. 2(c)]. In contrast, for *k* = 0, only one electron pass is required, taking less time to reach a similar gain level. That mechanism raises the detector’s gain meaning that the net number of the generated charges in the considered circuit per photocarrier pairs increases. That is a highly unwanted process to include the following reasons:

- time-consuming—limits the detector’s *BW*;
- random—increases the detector’s noise;
- unstable—leading to the avalanche breakdown.

It must be stressed that for materials exhibiting comparable multiplication coefficients and negligible “*dead space*” effect, although the breakdown prospect raises more slowly with voltage the breakdown process was found to be quick and jitter low. As the thickness of the multiplication area is scaled, the breakdown time and jitter decrease leading to time performance improvement which was confirmed for InP and Si SPADs. Moreover, an increase of the carrier’s velocities multiplicating in their tracks is believed to lower breakdown time and jitter^19^.

Figure 2(d) shows the carrier’s multiplication coefficients dependence on *E* (electric field) for selected materials used for the APDs’ fabrication. As can be seen, starting from *E* ~ 10^5 ^V/cm, the ionization coefficients raise rapidly versus a small *E* gradient, but for fields *E* < 10^5 ^V/cm carrier multiplication is insignificant for considered materials. For some materials to include: Si, GaAsSb, and InGaAs (where *α*_*e*_ > *α*_*h*_) electrons ionize more effectively than holes while for Ge, GaAs (where *α*_*h*_ > *α*_*e*_) holes multiplicate more efficiently than electrons.

Taking the above conditions into consideration, the APDs’ fabrication process requires materials allowing multiplication by either electrons or holes. When electrons exhibit a higher avalanche coefficient, the multiplication mechanism should be initiated by injecting the photogenerated electron at the p-type edge of the depletion layer. In that case, the material should exhibit as low as possible *k*-values. On the other hand, if holes launch the multiplication process, the photogenerated hole should be transported into the n-type edge of the depletion region assuming as high as possible *k*-values. The perfect single-carrier avalanche mechanism is reached when the following conditions are met:

- *k* = *α*_*h*_/*α*_*e*_ = 0 (*α*_*h*_ « *α*_*e*_) for electrons;
- *k* = ∞ (*α*_*h*_ » *α*_*e*_) for holes.

The impact ionization factor *k* also affects the *GBW*. The time needed for the APD to reach a required gain level is termed by the avalanche build-up time or multiplication time being inversely proportional to the *GBW*.

**Fig. 3:**
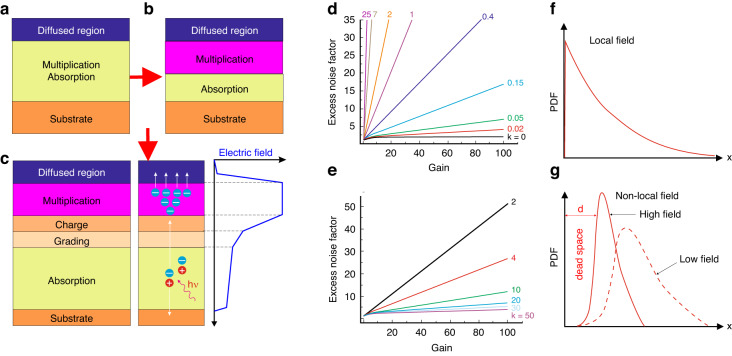
APDs. **a** *p*–*n* device, **b** SAM device, and **c** SAGCM device with electric field distribution. *F(M)* dependence on *M* for the selected *k* = *α*_*h*_/*α*_*e*_ in APDs when: **d** electrons and **e** holes dominate in the avalanche mechanism. The multiplication path length probability distribution functions in the: **f** local and **g** non-local field “*dead space*” models

Figure 3(a–c) show the InGaAs APDs design’s evolution. Initially, APD was designed as a *p*–*n* junction operating primarily in linear mode as shown in Fig. 3(a)^20^. Its operating bias was lower than multiplication breakdown voltage and the avalanche current scaled linearly to the light power. The limitation of the APD based on the *p*–*n* junction is that the depletion area containing the multiplication region is part of the absorption layer resulting in an electric field drop over both absorber and avalanche regions. Consequently, the APDs based on the *p*–*n* junction are characterized by high dark current (significant contribution of tunneling current is observed) and low gain. To remove this drawback, in 1979 Nishida et al. fabricated the detector with the n-InGaAsP absorption and P^+^-InP multiplication areas being separated by an extra n-InP layer (SAM) as presented in Fig. 3(b)^21^. Further evolution of the APD architecture occurred in the early 1990s by the implementation of isolated absorption, grading, charge, and multiplication structures (SAGCM) which allowed to suppress the tunneling current contribution [see Fig. 3(c)]^22,23^. The band offset between the absorber, avalanche layers, and design of the charge/grading regions have to be considered as the key parameters of the SAGCM. An additional n-InP charge region was introduced into the SAM structure to modify the distribution of the electric field. Furthermore, the InGaAsP grading area was added to reduce the valence band discontinuities between the InP and InGaAs regions. This resulted in avalanche structures with the highest sensitivity (SAGCMs) currently used in the NIR band [see Fig. 3(c)].

A complete theory of the APD’s multiplication excess noise was proposed by McIntyre^24,25^. This theory is created on the local-field model according to which the carriers’ multiplication coefficients are in equilibrium. The APD’s noise per unit bandwidth can be given by the equation:

2 $$\left\langle {I}_{n}^{2}\right\rangle =2q{I}_{{ph}}{\left\langle M\right\rangle }^{2}F(M),$$

where: <*M*>—average avalanche gain, *q*—electric charge, *I*_ph_—photocurrent for gain, *M* = 1, and *F(M)*—excess noise factor related to gain arising from the probabilistic character of the ionization effect.

According to the McIntyre theory, if the electrons initiate multiplication the *F(M)* may be calculated based on the following formula:

3 $${F}_{e}\left({M}_{e}\right)=k\left\langle {M}_{e}\right\rangle +\left(1-k\right)\left(2-\frac{1}{\left\langle {M}_{e}\right\rangle }\right),$$

while for the holes starting the avalanche process, the equation assumes:

4 $${{F}_{h}\left({M}_{h}\right)=\frac{1}{k}\left\langle {M}_{h}\right\rangle +\left(1-\frac{1}{k}\right)\left(2-\frac{1}{\left\langle {M}_{h}\right\rangle }\right)},$$

In terms of the simple p–n-based photodetectors under reverse voltage exhibiting no multiplication gain, <*M*> = 1, *F(M)* = 1 and the shot noise given by equation $\left\langle {I}_{n}^{2}\right\rangle =2q{I}_{{ph}}$ limits the detector’s performance. Assuming, that injected photocarriers exhibit the same gain *M*, *F(M)* = 1 and the noise power may be given by the noise caused by accidental transport of photogenerated carriers, multiplied by *M*^2^. In contrast, the multiplication effect is inherently stochastic, meaning that the carriers exhibit different avalanche gains distributed with mean gain <*M*>. This is related to the extra noise source referred to as avalanche over-noise, being easily given by the *F(M)* in Eq. (2). Figure 3(d, e) presents the APD’s *F(M)* versus *M* for the selected *k* = *α*_*h*_/*α*_*e*_. If *k* = 0 (pure electron injection) the *F(M)* maintains constant value versus gain, as presented in Fig. 3(d), while in terms of the pure hole injection observed for *k* > 50, *F(M)* stays constant versus *M* and changes for low *k* as presented in Fig. 3(e).

As already mentioned, to reach a low excess noise factor, the carrier’s ionization coefficients must be as different in values as viable, and the multiplication effect has to be launched by carriers with higher ionization coefficients. Most A^III^B^V^ semiconductors have an ionization factor within the range of 0.4 ≤ *k* ≤ 2^26^.

The local field model correctly describes the multiplication process and excess noise when the avalanche layer is thick (>1 μm). Figure 3(f, g) presents the carriers’ ionization probability in the avalanche region. Ionization probability decreases exponentially versus distance from the injection region, however, with thinning the multiplication region to the submicron level, the local field theory does not justify the *F(M)* lowering^27,28^. To explain this device phenomenon, a non-local effect in the multiplication mechanism was proposed^29,30^. The multiplication process is non-local and carriers transported into the high-electric field area need a specific length, to reach the necessary energy to multiplicate^29,31^. That specific length where carriers are not multiplicated is called “*dead space*”, *d*. The “*dead space*” effect imposes the changes in the probability distribution function (PDF) of the multiplication effect as presented in Fig. 3(g). For the thin multiplication region, the electric field must be higher than assumed to reach a specific impact ionization increase. When the “*dead space*” is considered, the PDF width is narrower causing the multiplication mechanism more deterministic.

Therefore, *F(M)* can be suppressed by thinning (scaling) the impact ionization layer. The “*dead space*” for both carriers could be roughly estimated by *E*_*th*_—ionization threshold energy depending on the material’s band structure and *E* (~*E*_*th*_/*qE*). The “*dead space*” contribution may be substantial leading to significant excess noise suppression due to a much narrower PDF than given by the local field theory. Consequently, an APD exhibiting low excess noise may be fabricated based on material exhibiting *k* ~ 1^2,16,32^. The avalanche region length reduction is another advantage—it increases the frequency response.

The APDs *GBW* is derived from the time needed for the multiplication effect to decay or build up. The time constant, gain, and bandwidth are related to each other. The lower bandwidth the higher gain and the higher the time constant, however, it was Emmons who presented that the bandwidth limitation disappears when either electron or hole ionization coefficients assume *α*_*h*_ = *α*_*e*_ = 0^33^. Assuming non-zero ionization coefficients (*α*_*h*_ ≠ 0, *α*_*e*_ ≠ 0), the time dependence of the average electron-initiated gain may be estimated by the equation:

5 $$M\left({\rm{\omega }}\right)=\frac{{M}_{o}}{\sqrt{1+{\left({\rm{\omega }}{M}_{o}k{\rm{\tau }}\right)}^{2}}},$$

where: *M*_*o*_ is the DC gain, *τ* is roughly the carrier transit time across the avalanche layer.

As is marked above, the most important APD performance could be given by:

- excess noise factor [*F(M)*];
- bandwidth (*BW*);
- gain (*M*);
- gain-bandwidth product (*GBW*).

Three approaches to designing and fabricating high-performance APDs could be distinguished to obtain low *F(M)* and high *GBW*:

- semiconductor selection exhibiting proper impact carrier multiplication coefficients;
- thinning the avalanche area to use the multiplication effect non-local field capability;
- properly designed heterojunctions by impact ionization engineering (*I*_*2*_*E*).

The current bulk and type-II superlattice (T2SLs) materials suitable for high-performance APDs’ fabrication and their spectral ranges are gathered in Table 1. In turn, Table 2 compares their general state-of-the-art to include cut-off wavelength (*λ*_*c*_), quantum efficiency (*QE*), gain (*M*), excess noise factor [*F(M)*], operating temperature (*T*), manufacturability, and limitations with technology readiness level (TRL)^34^.

**Table 1:** Materials used for high-performance APDs

Material	Spectral range	General characteristics
Si	0.4–1.1 μm	• hole ionization rate much lower than electron ionization coefficient (*α*_*e*_ » *α*_*h*_)
Ge	0.8–1.65 μm	• bandgap is smaller than Si • hole and electron ionization comparable rates (*α*_*e*_ ≈ *α*_*h*_) • Ge-based APDs exhibit high excess noise (limited applications)
GaAs-based	below 0.9 μm	• *α*_*e*_ ≈ *α*_*h*_ for most compounds • *α*_*e*_(GaAs) » *α*_*e*_(AlGaAs) • typical heterostructure: GaAs/Al_0.45_Ga_0.55_As • high gain caused by the multiplication mechanism in GaAs layers • applying InGaAs layers extends sensitivity to ≈ 1.7 μm
InP-based	1.2–1.6 μm	• low-excess noise lattice-matched heterostructure n^+^-InP/n-GaInAsP/p-GaInAsP/p^+^-InP—most carriers transported into high *E* area • p^+^-InP/n-InP/n-InGaAsP/n^+^-InP heterostructure (comparable to Si) • absorption in InGaAsP region and minority carriers avalanche multiplication occurs in n-InP region
Hg_1–*x*_Cd_*x*_Te	1–12 μm	• electron-initiated multiplication demonstrated for Cd composition, *x* = 0.7–0.21 • avalanche gain ~100 provides 10–20× lower noise than InGaAs or InAlAs, 4× lower noise than Si APDs
Type-II superlattices (T2SLs)	1–12 μm	• believed to reach lower noise and higher *M* and breakdown bias than HgCdTe APDs • superior high gain performance is conditioned on the flexibility in band structure engineering suited for much higher electric fields than HgCdTe

**Table 2:** Avalanche photodiodes based on selected materials—state-of-the-art

Material	Cut-off wavelength [μm]	Quantum efficiency	Gain ≥ 500	Excess noise ≤2	Operating temperature ≥200 K	Manufacturability	Limitations
Si	1.1	Low	Yes	Yes	Yes	High	Spectral coverage
InGaAs/InP	1.67	High	No	No	Yes	High	Spectral coverage, Limited gain
Extended InGaAs	2.5	High	No	No	Yes	Low	Limited gain, Manufacturability
HgCdTe	5	High	Yes	Yes	No	Low	Operating temperature, Manufacturability
InAs	3.8	High	Yes	Yes	No	Med	High tunneling dark current, No cooling advantage
AlInAsSb on GaSb	2	Low	Yes	Yes	Yes	Med	Low *QE*, Growth challenges, Low TRL^a^
AlGaAsSb and AlInAsSb on InP	> 2	High	Yes	Yes	Yes	High	Low TRL^a^

^a^*TRL* technology readiness level

## A^III^B^V^ infrared avalanche photodiodes

The semiconductor’s selection for APDs fabrication is conditioned by applications where the most common are fiber optic communications, high-speed receivers, single-photon counters, and laser range finders. Even though the IV-group semiconductor materials such as Si and Ge exhibit superior performance among APDs, Si and Ga-based APDs cannot operate in a 1.55 μm optical communication band due to their cut-off wavelength limitations. For this reason, the research efforts have been directed at InGaAs/InP APDs. Much current research on the APDs has been focused on the development of the new architecture and the materials substitutions/alternatives to lower dark current, to reach higher speed and lower excess noise maintaining optimal gain levels at the same time. Recently GeSn APDs have been introduced to circumvent the longer cut-off wavelength limitations^35,36^. The InAlAs or InP submicron multiplication areas with InGaAs absorption layers (InGaAs is reported to be lattice matched to InAlAs and InP) could be used to reach lower *F(M)* because of “*dead space*” effect. The InAlAs *k* = *α*_*h*_/*α*_*e*_ is reported to be much higher than the InP *k* reached for low *E*. The InAlAs *F(M)* is much lower than in InP at a given gain due to the high InAlAs *α*_*h*_/*α*_*e*_ ratio and the favorable InP “*dead space*” effect. Moreover, light with a wavelength, *λ* > 1.4 μm called “*eye-safe*”, goes to the eye anterior potions eye (primarily the cornea) consequently not reaching the retina. Since Si does not absorb beyond > 1 μm, A^III^B^V^ semiconductors offer the potential for longer wavelengths of LIDARs.

**Fig. 4:**
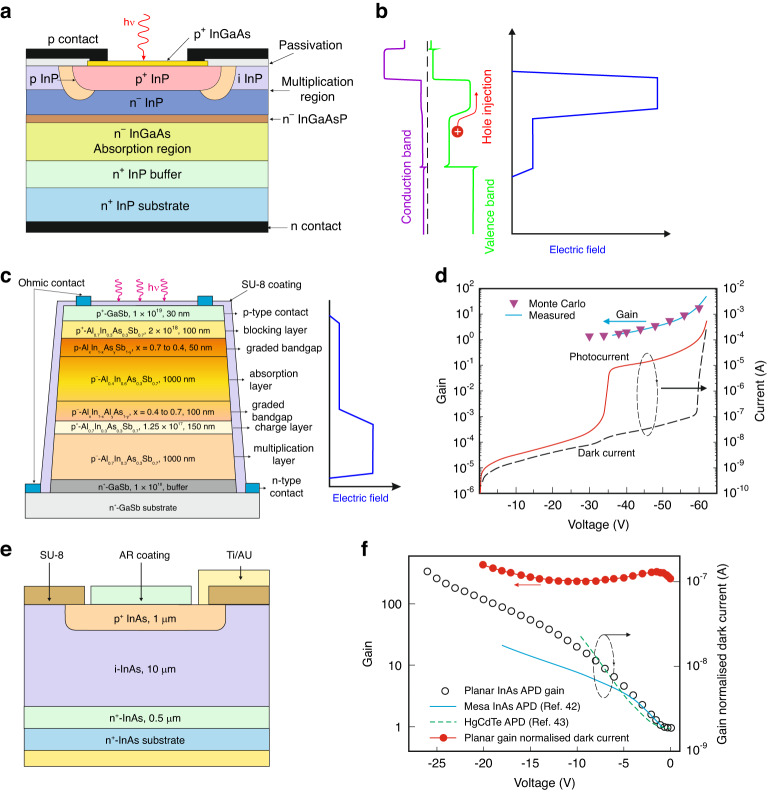
InGaAs/InP SAM-APD. **a** device structure, **b** energy band profile, and electric field under normal reverse bias condition. Al_*x*_In_1–*x*_As_*y*_Sb_1–*y*_ based SACM APD: **c** detector’s design with the *E* distribution within the detector, **d** measured and theoretically simulated gain, dark current, photocurrent versus reverse voltage for 90 μm diameter device at room temperature^39^. InAs planar avalanche photodiode: **e** a schematic design diagram, **f** comparison of the gain reached by 1550 nm wavelength laser^132,133^. The *M* normalized dark current for 100 μm radius planar APD was presented for 200 K

For high-speed telecommunication receivers, the APDs exhibiting short response time and high *GBW* are required. Time response and *GBW* are mainly restricted by the profile of the heterojunction between the active and avalanche regions and the doping distribution within the detector. Attempts to reach improvement in avalanche gain for InGaAs by rising the electric field are not feasible which is related to the tunneling effects resulting in high leakage currents. The low value of the electron effective mass causes a sharp increase in tunneling current for electric fields, *E* > 150 kV/cm^37,38^. That drawback was circumvented by combining an InGaAs absorber layer operating with low *E* and a lattice-matched InP multiplication layer with a wider bandgap responsible for impact ionization. That architecture is an example of the previously mentioned SAM-APD design (isolated absorption and multiplication layers). The InGaAs/InP SAM-APD device structure, with a double-diffused floating guard ring, is presented in Fig. 4(a), while the heterostructure energy band profile and electric field distribution are presented in detail in Fig. 4(b). The radiation is absorbed in InGaAs active layer and photogenerated holes [exhibiting higher multiplication coefficient than electrons which guarantees low *F(M)*] are transported to InP heterojunction, where the impact ionization occurs. That design allows for low surface current caused by the junction being located in the wide energy gap InP providing responsivity in the longer wavelength range by the low energy gap InGaAs active layer.

InGaAs/InP heterojunction APDs are usually built of the 1–2 μm thick undoped active layer. The 0.1–0.3 μm thick InGaAsP grading and 1–2 μm thick multiplication regions are doped up to the level 1 × 10^16^ cm^−3^. The p^+^-layer is thin and doped up to the level of 10^17^–10^18 ^cm^−3^. The junction is typically produced by Zn p^+^-type diffusion into the InP avalanche region and Cd diffusion (or implantation) for the guard ring into the top InP layer through the SiO_2_ mask.

It must be stressed that the valence band offset at the InGaAs/InP junction accumulates holes in the valence band which deteriorates the device’s response time. That valence band discontinuity is reduced by grading the bandgap of the quaternary InGaAsP layer grown between the InP and InGaAs regions. That improved architecture is referred to as the separate, absorption, graded, multiplication avalanche photodiode (SAGM).

The Al_*x*_In_1–*x*_As_*y*_Sb_1–*y*_ separate absorption, charge, and multiplication (SACM) APD is presented in Fig. 4(c). The device architecture includes (order from the very top): GaSb contact layer, p-type Al_0.7_In_0.3_As_0.3_Sb_0.7_ (100 nm thick, 2 × 10^18 ^cm^−3^) blocking region, Al_*x*_In_1–*x*_As_*y*_Sb_1–*y*_ within the region *x* = 0.4–0.7 grading region, p^−^-type Al_0.4_In_0.6_As_0.4_Sb_0.4_ 1000 nm thick active region, 150 nm thick, p^+^-type Al_0.7_In_0.3_As_0.3_Sb_0.7_ charge region (1.25 × 10^17 ^cm^−3^), 1000 nm thick p^−^-type Al_0.4_In_0.6_As_0.4_Sb_0.4_ multiplication region, and finally n-type GaSb contact region [(1–9) × 10^17 ^cm^−3^]. Lastly, N_2_/Cl_2_ inductive coupled plasma (ICP) and typical photolithography with bromine methanol and SU-8 treatment to reduce leakage current were used to define circular mesas.

Under the strong reverse bias, the high *E* within the multiplication layer enables the avalanche effect, and photogenerated electrons drift is realized by a small electric field suppressed by the charge region in the absorber layer. Figure 4(d) shows 25 μm radius Al_*x*_In_1–*x*_As_*y*_Sb_1–*y*_ SACM APD performance. The dark current at 95% breakdown voltage assumes ~120 nA, being roughly ~100× lower than the current for APDs based on Ge on Si and like AlInAs/InGaAs APDs^39^. The experimental *M* ~ 50 values were confirmed by the Monte Carlo simulations.

In the last decade, a new breakthrough in the development of InAs APDs has been reached^40^. Their high potential is conditioned by the low production expenses related to the easily available A^III^B^V^ fabrication foundries and the relatively low price of the 6” native substrates, as well as the operation using thermoelectric cooling. The historical problem with the surface leakage of InAs photodiodes is gradually reduced by elaborating wet chemical etching recipes like the solutions of phosphoric and sulfuric acid-based etchants^41^.

Both mesa and planar InAs APDs were fabricated. For mesa p-i-n structures, the *M* normalized dark current density (*J*_Dark_) at the level of ~5 × 10^−6^ A/cm^2^ for LN_2_ temperature (77 K) has been published^42^. The onset of BTB tunneling at moderately low *E* requires a thicker multiplication region to achieve high multiplication gain exacerbating passivation difficulties. To resolve that issue, planar structures as shown in Fig. 4(e) have been developed and described in detail in ref. ^40^. To form a *p*–*n* junction, Beryllium (Be) implantation at low energy was used.

Table 3 presents the APDs based on Si, Ge, and InGaAs parameters/performance. Data is shown for comparison reasons among materials used for APDs’ fabrication.

**Table 3:** Review of Si, Ge, and InGaAs APDs’ performance

Performance	Si	Ge	InGaAs
Wavelength (nm)	400–1100	800–1650	1100–1700
Peak wavelength (nm)	830	1300	1550
Current responsivity (A/W)	50–120	2.5–25	–
*QE* (%)	77	55–75	60–70
*M*	20–400	50–200	10–40
*J*_*Dark*_ (nA)	0.1–1	50–500	10–50 (*M* = 10)
Rise time (ns)	0.1–2	0.5–0.8	0.1–0.5
*GBW* (GHz)	100–400	2–10	20–250
Voltage (V)	150–400	20–40	20–30
Capacity (pF)	1.3–2	2–5	0.1–0.5

Progress in materials’ properties and advanced detector structures have increased the APDs performance for fiber optic communication systems over the past decade^16,17,43,44^. These include the introduction of the continuous or grading bandgap for absorption/avalanche layers to limit carrier trapping and insertion of the electric-field control layers. Advanced APDs structures require the multiplication region thickness to be shrinked to reach fast response times. The local McIntyre theory does not properly justify the excess noise characteristics for the devices with thin multiplication regions. The InP APD with the 0.25 μm-thick multiplication region reaches the excess noise performance scaling with 1/*k* ~ 0.25 for the hole-initiated avalanche process (h-APD), however, according to the McIntyre model, the multiplication factor is reported at the level of ~0.7. The significant reduction in excess noise can be reached by the “*dead space*” effect in the thin multiplication layers. Additional improvements in the detector’s excess noise may be reached by the implementation of the InAlAs/InAlAsSb (instead of InP) being lattice-matched to InGaAs and InP as the multiplying layer. The InAlAs/InAlAsSb *α*_*h*_/*α*_*e*_ was estimated to be much higher than the InP *α*_*h*_/*α*_*e*_ ratio at low *E*. The InAlAs *F(M)* at a given *M* is much smaller than in InP being related to the high InAlAs *α*_*h*_/*α*_*e*_ ratio and the favorable “*dead space*” effect in InP.

**Fig. 5:**
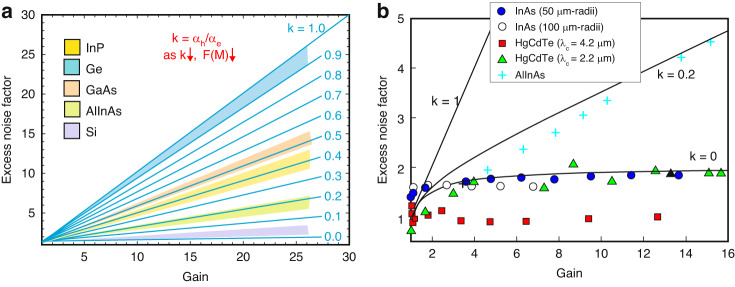
*F(M)* versus *M* for. **a** Si, AlInAs, GaAs, Ge, InP [the solid lines present the *F(M)* for *k* within the range 0–1 (increment 0.1) calculated by the local field model^24^, typical *F(M)* are shown by shaded regions^37^ and **b** selected materials: 3.5 μm thick intrinsic InAs APDs (50 μm and 100 μm radius), 4.2 μm cut-off wavelengths HgCdTe and 2.2 μm InAlAs APDs^134^

Figure 5(a) presents the *F(M)* versus *M* for selected material systems. The solid lines present the *F(M)* for *k* = 0–1 values simulated by the local field theory^24^. In general, *F(M)* should increase versus *k*. Typical excess noises are shown by shaded regions^37^. The *k* = *α*_*h*_/*α*_*e*_ values for the best commercially available Si APDs stay within the range 0.01–0.06. InP and InAlAs commonly implemented as avalanche regions of the APDs for telecommunication applications assume higher *k*-values:

- InP within the range *k* = 0.4–0.5;
- InAlAs within the range *k* = 0.2–0.3^44^.

Lately, two quaternary A^III^B^V^ bulk compound semiconductors, Al_*x*_In_1–*x*_As_*y*_Sb_1–*y*_ to GaSb and Al_x_Ga_1–*x*_As_*y*_Sb_1–*y*_ lattice-matched to InP were reported to reach excess noise comparable to Si^17^. The Al_*x*_Ga_1–*x*_As_*y*_Sb_1–*y*_ APDs within the range of Al chemical composition, *x* = 0.5–0.7 exhibit *k*-values at the level of 0.01. This behavior is explained by the significant domination of electron impact multiplication in comparison to the holes, which is related to the Sb contribution/content. It is suggested that Sb-content increases photon scattering rates and increases effective hole mass causing a significant suppression of hole ionization coefficient, *α*_*h*_. Both Al_x_Ga_1–*x*_As_*y*_Sb_1–*y*_ and Al_*x*_In_1–*x*_As_*y*_Sb_1–*y*_ quaternary compounds are considered to have a potential for ≥2 μm wavelength optical communication band. Figure 5(b) shows *F(M)* versus *M* for InAs APDs compared with data for selected materials: HgCdTe and InAlAs. The *F(M)* values estimated for InAs p-i-n avalanche photodiodes do not follow McIntyre theory falling below the local field model assessed for *k* = 0 being similar to the reported for SWIR HgCdTe and slightly higher than published for MWIR HgCdTe electron-initiated, e-APDs. This *F(M)* dependence on gain falling under the lower limit of the local field theory is related to “*dead space*” effect. As marked in the section “Background”, if the avalanche layer is thick, “*dead space*” may be ignored, and McIntyre local field theory correctly justifies the APD’s performance. The InAlAs APD *F(M)* dependence on *M* is comparable to the standard APDs where both electrons and holes undergo multiplication.

The progress in InAs electron-initiated, e-APDs’ fabrication allowed the ideal properties of avalanche multiplication and excess noise to transfer to the readily available A^III^B^V^ materials system, enabling broader applications that were previously only possible with the less accessible HgCdTe system. The InAs APDs properties make them an attractive approach for a wide range of NIR and MWIR purposes, including active/passive imaging, LIDAR, and remote gas sensing.

## A^II^B^VI^ avalanche photodiodes

**Fig. 6:**
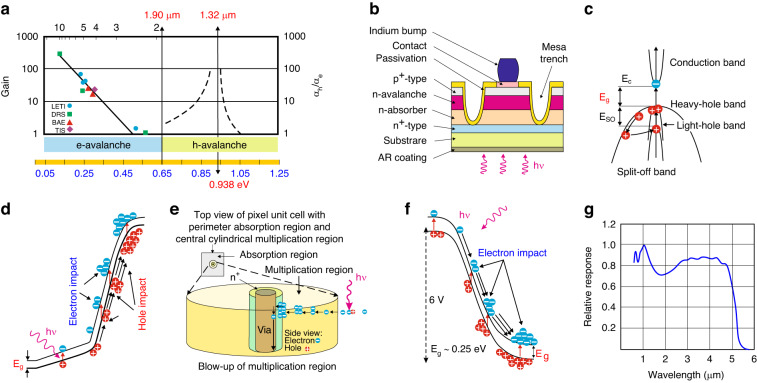
Gain and *k* versus Hg_1–*x*_Cd_*x*_Te bandgap energy. **a** the crossover between e-APD and h-APD. The crossover at *E*_*g*_ ≈ 0.65 eV corresponds to the *λ*_*c*_ = 1.9 μm for 300 K^46^. Hole-initiated avalanche HgCdTe photodiode: **b** detector profile, **c** energy band structure, **d** hole-initiated multiplication process energy band structure. The multiplication layer bandgap energy is adjusted to the resonance condition where the bandgap and the split-off valence band energy and the top of the heavy-hole valence band energy difference are equal. Electron-initiated avalanche HgCdTe photodiode: **e** diagram of electron-initiated avalanche process for HgCdTe-based high-density vertically integrated photodiode (HDVIP) structure (n-type central region and p-type material around), **f** electron avalanche mechanism, and **g** relative spectral response for 5.1 μm cut-off wavelength HgCdTe HDVIP at *T* = 80 K

As reported by Leveque et al. it is possible to distinguish two regions of the Hg_1–*x*_Cd_*x*_Te, *x* Cd chemical compositions where *k* = *α*_*h*_/*α*_*e*_ is either much higher *k* » 1 or much lower than *k* «1 what was presented in Fig. 6(a) showing avalanche Hg_1–*x*_Cd_*x*_Te capability dependence on the bandgap energy^45,46^. For a cut-off wavelength shorter than about *λ*_c_ < 1.9 μm (*x* = 0.65 for 300 K), authors estimated *α*_*e*_ « *α*_*h*_ due to the resonant enhancement of the hole multiplication coefficient when bandgap energy corresponds to the *E*_*g*_ ≅ *E*_*SO*_ = 0.938 eV [see Fig. 6(c)] what corresponds to the 1.32 μm. The case for *k* » 1 is favorable for low *F(M)* APDs with a hole-initiated multiplication effect. The electron-initiated multiplication effect is dominant for *x* < 0.65. Both HgCdTe *k* regimes could be used for efficient APDs utilizing comparable SAM device structures.

Figure 6(b–d) illustrates the APD device profile, energy band structure, and multiplication mode for hole-initiated avalanche (h-APD) HgCdTe photodiodes. In this case, the bandgap energy [*E*_*g*_, see Fig. 6(c)] corresponds to the energy difference between the top valence and the split-off light-hole band (*E*_*SO*_). Assuming the advantage of that regime, de Lyon et al. published on the back-illuminated multilayer SAM-APD deposited in situ by MBE on CdZnTe exhibiting *λ*_*c*_ = 1.6 μm and avalanche region, *λ*_*c*_ = 1.3 μm^47^. Multiplication gain within the ranges, *M* = 30–40 at *V* = 80–90 V reverse voltages for 25-element mini-arrays was demonstrated.

Originally, quite a few experimental papers were published to verify the predicted hole-to-electron impact ionization low ratios, *k* < 0.1 values for Hg_1–*x*_Cd_*x*_Te exhibiting cut-off wavelengths, *λ*_*c*_ > 1.9 μm^48^. In 1990, Elliott et al. predicted reasonable gain values, *M* ~ 5.9 at low reverse voltages, *V* = −1.4 V for electron-initiated LWIR HgCdTe APDs (*λ*_*c*_ = 11 μm)^49^. The very first strong and persuasive benefits of the electron-initiated multiplication mechanism in the MWIR lateral-collection n^+^-n^−^-p APDs (p-type active layer) were published by Beck et al. in ref. ^50^.

A concept presented by Kinch in his monograph (Chapter 7) explains in detail the high-value difference between electron and hole ionization coefficients (*α*_*e*_ ≠ *α*_*h*_) resulting from HgCdTe energy band diagram characteristics, including:

- hole effective mass higher than electron one (holes exhibit lower mobility);
- low optical phonons scattering rates;
- two times lower electron multiplication threshold energy^51^.

The electron-initiated HgCdTe APDs have been designed and fabricated by DRS, BAE Systems Infrared in England, and Sofradir/Leti in France^52–54^. The most popular APD structures are presented in Table 4. The DRS detector was referred to as an HDVIP, while BAE Systems Infrared reported on the loophole diode^55,56^. The avalanche process in HgCdTe HDVIP structure is illustrated in Fig. 6(e, f). The carriers are photogenerated in the p-type active region (surrounding the center n-type avalanche region) and then diffuse into the multiplication region. If the reverse voltage increases within the range from 50 mV to several volts, the central n-type multiplication layer comes to be completely depleted where a high-electric field builds up accelerating low effective mass electrons to avalanche in HgCdTe low bandgap multiplication material. As is presented in Fig. 6(g), the front side of the illuminated APD responds with high *QE* from the VIS to the IR cut-off wavelengths, however, due to the narrow bandgap energy of the compound building the avalanche layer, the APD requires severe cryogenic cooling.

**Table 4:** HgCdTe-based avalanche photodiode arrays

Device architecture	Spectral range	Device geometry	Performance	Ref.
HDVIP (DRS)	3–5 μm	128 × 128, pixel 40 μm	*M* > 1000, *F(M)* ~ 1.3, *J*_Dark_ < 1 nA/cm^2^, *BW* ~ 100 MHz	^102^
Mesa (Leonardo)	0.8–2.5 μm	320 × 256 pixel 24 μm	MOCVD growth on GaAs substrates, operating temperature, *T* = 90–100 K, *J*_Dark_ = 1–100 nA/cm^2^, *QE* ≈ 70%, *M* ≤ 400, *F(M)* < 1.3	^62^
Planar (Lynred/Leti)	2–5 μm	320 × 256, pixel 30 μm	MBE growth, *F(M)* ∼ 1.3–2.2 at *M* ∼ 10, *QE* ∼ 30–40%, diffusion current density ∼ 0.1–0.3 mA/cm^2^, operating temperature, *T* = 80 and 200 K	^103^
	3–5 μm	384 × 288, 15 μm pitch size, operating temperature, *T* ~ 80 K	MBE and LPE growth, the highest gain achieved, *M* ~ 13000 at *V* = −13.8 V, *F(M)* < 1.4; operability ~99.7%; HgCdTe FPA image with 1 μs integration time and *M* = 30.	^104^

**Fig. 7:**
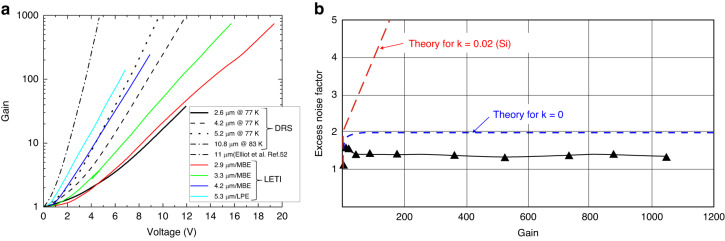
HgCdTe APDs performance. **a** the experimental gain versus bias for selected cut-off wavelengths for DRS electron-initiated APDs at 77 K together with extra measured data points taken at ∼77 K^51^ and LETI e-APDs at 80 K^59^, **b** constant *F(M)* ~ 1 versus *M* at 80 K for 4.3 μm cut-off wavelength APD^135^

Empirically determined electron multiplication gain (*M*_*e*_) for HgCdTe photodiodes at 77 K is equal:

6 $${M}_{e}={2}^{2\left(V-{V}_{{th}}\right)/{V}_{{th}}}+1,$$

with *V*_*th*_ ≈ 6.8 × *E*_*g*_ for all Cd compositions from 0.2 < *x* < 0.5^50^. Figure 7(a) shows the measured gain dependence on the bias, *V*, together with DRS experimental data. The DRS HDVIPs experimental data shows nearly “perfect” APD characteristics/performance. The detector exhibits the homogenous exponential gain versus bias characteristic being consistent with *k* = *α*_*h*_/*α*_*e*_ ≈ 0. The *F(M)* data for photodiodes with a 4.3 μm cut-off wavelength shows no dependence of *F(M)* on *M* where *F(M)* = 1.3 for *M* > 1000 [see Fig. 7(b)], proving that the electrons undergo the ballistic ionization process^57,58^. The high bandwidth large area pixels can be reached by joining the APDs with small capacitance in parallel (N × N configuration) due to the cylindrical junction geometry.

More recently, there have been reports on other device structures confirming the important features of the electron-initiated multiplication mechanism—see Table 4 where schematic illustrations of the mesa heterojunction and planar homojunction are presented. Selex in Southampton (at present Leonardo) designed and fabricated the mesa heterojunctions (grown by MOCVD on GaAs substrates) with the energy bandgap and doping levels to be varied easily within the detector’s architecture. Both the absorber and avalanche regions are individually adjusted. Every pixel is electrically screened by a mesa slot extending through the active layer to suppress lateral-collection and blooming.

The HgCdTe Leti/Sofradir planar p–i–n homojunctions with sizable n-regions are processed by the n-type conversion. The vacancy-doped p-type (*N*_*a*_ = 3 × 10^16 ^cm^–3^) thin layer next to the surface is converted into a n^+^ type to the doping level, *N*_*d*_ = 1 × 10^18 ^cm^–3^^54^. During processing of the n^+^ type doping, n^−^ layer is formed by the Hg vacancies reduction to the typical epitaxy residual doping level *N*_*d*_ = 3 × 10^14 ^cm^–3^. The broadening of the lightly doped n^−^ layer is associated with the thickness of the highly doped n^+^ region.

The highest gain-bandwidth product, *GBW* > 16 THz was reported by Leti/Sofradir APDs^59^. Figure 7(a) shows typical gain curves reached and presented by LETI for selected electron-initiated APDs versus cut-off wavelengths at 80 K.

Perrais et al. reported on the utmost gain, *M* = 5300 at *V* = −12.5 V for MBE-grown 30 μm pitch p-i-n HgCdTe planar APD deposited on a CdZnTe with 5 μm cut-off wavelength^60^. As shown in Fig. 7(a), the utmost *M* normally follows an exponential trend with reverse bias and cut-off wavelength.

The standard performance of the HgCdTe avalanche photodiode at temperature 80 K is presented in Table 5^61^. The highest gains stay within the range from 2000 for SWIR photodiodes up to 13,000 in MWIR devices and agree with the maximum stable gain values. Those such high gain values depend on the APD’s observation time, dark current noise, and the noise of the detection electronics. The SWIR APDs are characterized by a stable gain related to the low noise, up to 300 K.

**Table 5:** SWIR and MWIR HgCdTe APD performance at 80 K

Performance	SWIR	MWIR
*QE*	60–80%	
Max gain, *M*	2000	13000
Voltage at *M* = 100	12–14	7–10
*F(M)*	1.1–1.4	
*QE* to *F(M)* ratio	40–70%	
Response time *T*_90–10_	0.5–20 ns	
Max *GBW*	2.1 THz	

Electron-initiated HgCdTe APDs allow additional advantages for the focal plane arrays (FPAs) fabrication for SWIR and MWIR ranges. These detectors are being used for gated-active/passive imaging—see section “Avalanche photodiodes in active imaging systems”. Table 4 collects the performance of the most advanced HgCdTe APD FPAs. The first demonstration of 24 μm pitch APD 320 × 256 laser-gated imaging FPA was reported by Baker et al. in Selex^53^. Selex reported on the 4.2 μm cut-off wavelength APDs exhibiting multiplication gains up to 100, low excess and input noises being equal to the photon noise at the level of 15 photons rms for 1 μs integration times. Lately, Selex and Leti have designed and fabricated those devices for space purposes^61,62^. Selex has also reported on the full-custom silicon read-out integrated circuit (ROIC) for SAPHIRA (Selex Advanced Photodiode Array for High Speed Infrared Array). That 24 μm pixel pitch 320 × 256, FPA is developed for wavefront sensors and interferometry applications in the space telescopes, and its specification and performance are included in Table 6. The present version of SAPHIRA FPAs has exhibited sensitivity within the range 0.8–2.5 µm, *QE* > 80%, short-time response, *M* > 500, and sub-electron effective read noise (~0.1e^−^ rms) at 1 kHz frame rate and operating temperatures, *T* = 90–100 K^63^.

**Table 6:**
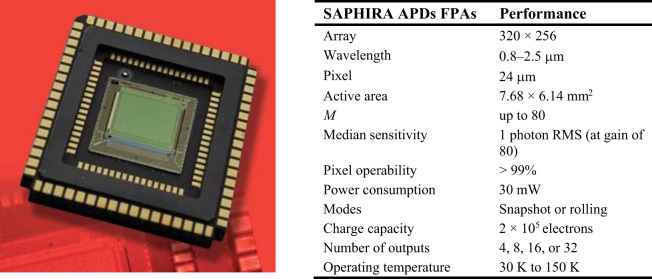
SAPHIRA APDs FPAs performance

The University of Hawaii together with partners (Leonardo corporation, Markury Scientific, and Hawaii Aerospace) driven by the SAPHIRA performance has started to develop a 15 μm pixel 1k × 1k FPA appropriate for ultra-low background IR space applications, reaching dark current, *J*_Dark_ < 0.001 e^−^/pix/s and a read noise <0.3 rms e^−^/pix/frame^64^.

The French company, First Light Imaging developed the C-RED One camera with SAPHIRA detector developed by Selex. The camera is cryogenically cooled by an integrated pulse tube. The latest version of the camera with an *f*/4 beam aperture is characterized by a dark current induced by a blackbody at 80 K of 30–40 e^−^/s at a gain, *M* = 10^65^.

## Superlattice avalanche photodiodes

The APDs’ noise may be suppressed not only by the selection of the materials exhibiting high ionization coefficients but also with thin/scaled multiplication regions. Further suppression is expected and confirmed by the implementation of the new materials (“*third wave*”) and impact ionization engineering (*I*_*2*_*E*) with correctly constructed and fabricated structures. The *I*_*2*_*E* architectures that have reached the lowest *F(M)* use avalanche layers where carriers are transported from a wide energy gap material to adjacent low bandgap semiconductors.

**Fig. 8:**
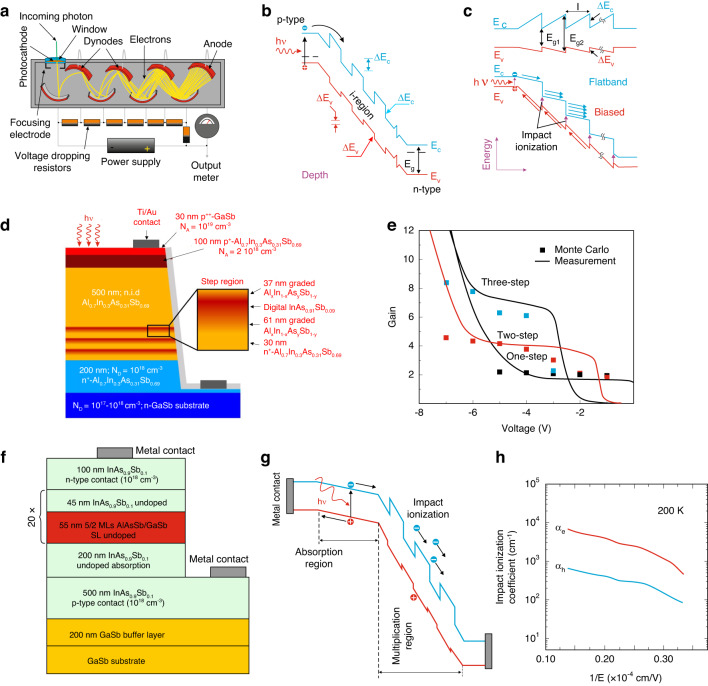
The device structure comparison between low-noise PMT and multi-quantum well APDs. **a** schematic presentation of a photomultiplier tube, **b** multi-quantum well p-i-n APD energy band sketch with marked intrinsic region (i), **c** energy band profiles of staircase APD under zero (top) and reverse (bottom) voltage. Multistep AlInAsSb staircase avalanche photodiode: **d** 3-step staircase APD device profile, **e** theoretically calculated by Monte Carlo method and measured gain of 1-, 2-, and 3-stairs APDs for 300 K^70^. MWIR SAM-APD structure with AlAsSb/GaSb superlattice: **f** device design profile, **g** energy band structure under reverse voltage, and **h** carriers impact multiplication coefficients versus reciprocal electric field at 200 K^73^

Prior to the development of bulk-based APDs, photomultiplier tubes (PMTs) were considered to be the preferred detector family for ultraviolet (UV) and NIR applications. Those detectors convert impinging photons to electrons on a photocathode and electrons are multiplied via a series of dynodes to a final anode [see Fig. 8(a)]. The arrival of an electron causes additional electrons to be released by every dynode producing high gain being scaled by a number of dynodes and the bias deposited on dynodes. PMTs are reported still to be used for some purposes, mainly due to their high sensitivity. On the other hand, PMTs are colossal, unstable, and require extremally high voltage which limits their potential applications.

The considerations in the section “Background” suggest that discrete localization of impact ionization effects and single-carrier multiplication are needed for the APD to reduce noise. The APD’s frequency response is conditioned by the carrier avalanche mechanism and the transit time, with the frequency response mostly being higher than the transit time due to the multiplication build-up time. In turn, the multiplication build-up time is conditioned by the electrons and holes ionization rates. Considering a heterojunction system with different conductivity and valence band edge discontinuities, the electron multiplication rates may be increased.

In 1982, for the first time, Capasso et al. presented the APD model simulating the functionality of a PMT^66,67^. This idea was examined in the multi-quantum well AlGaAs/GaAs APD shown in Fig. 8(b). Due to the fact that the conduction band offset (CBO) is higher than that at the valance band offset (VBO), the electron multiplication coefficient is higher than holes at the heterojunction interface. Further investigations showed that there is a lack of enough CBO and energy difference between the direct and indirect valleys of GaAs/AlGaAs for the staircase gain mechanism. In general, however, the above proposal has not been limited to GaAs/AlGaAs and other semiconductor systems have been widely used so far^68^. The biased staircase design shown in Fig. 8(c) prompts the single-carrier, electron-initiated avalanche process, while the hole-initiated multiplication is restricted by the lack/(small) of valence band discontinuity.

More recently published papers have shown that AlInAsSb/GaSb staircase APD with a near-ideal gain of 2 per stair allows to reach highly deterministic and low-noise operation^69,70^. Fig. 8(d) presents the schematic profile of a three-step AlInAsSb staircase APD and a demonstration of deterministic ~2^*n*^ gain dependence (*n*—number of stairs). As is shown, the stepped regions are composed of digital alloy grading between Al_0.7_In_0.3_As_0.31_Sb_0.69_ and InAs_0.91_ Sb_0.09_. The both bottom and top of the mesa structure are doped at moderate levels of acceptor and donor concentrations to form contact regions. Within the upper 600 nm of the mesa, generated electrons reach the energy by diffusion in the p-contact layer and *E* drift in the uniform Al_0.7_In_0.3_As_0.31_Sb_0.69_ unintentionally doped region. The electric field dropped on the stepped region allows electrons (discrete multiplication process) to gain enough energy for low-noise collision ionization^69^. As can be seen, the gain is multiplied from zero to ~2^*n*^ with reverse bias increasing to the level that all device stages reach a stepped state. At higher reverse voltage the gain increases above 2^*n*^ which is caused by band-to-band tunneling through InAs_0.91_Sb_0.09_ energy gap in the stages. Monte Carlo simulation results coincide well with the experimental data presented in Fig. 8(e) where to gain for 1-, 2-, and 3-stairs devices reach 1.77, 3.97, and 7.14 being comparable with numerically estimated values 2.01, 3.81, and 6.71.

Type-II superlattices (T2SLs) meet the bandgap requirements for APDs’ fabrication exhibiting high performance to include gain and low noise, and a single or dominant electron- or hole-initiated avalanche process in SWIR and MWIR ranges^71–74^. The “Ga-based” SLs have much larger VBO and CBO than the InAsSb layers in the “Ga-free” T2SLs. The T2SLs energy bandgap is conditioned by the SLs period and the Sb chemical fraction. Varying the width of the layer, C_1_ may be positioned between the InAs and GaSb conduction bands (CBs), while HH_1_ may be placed between their valence bands (VBs). The C_1_ band is more sensible to layer width than HH_1_ caused by the high GaSb heavy-hole mass (~0.41 *m*_*o*_). It was proved that the GaSb layer width has negligible influence on the T2SL energy bandgap, but due to the tunneling of InAs electron wave functions via GaSb barriers, the GaSb width significantly contributes to the conduction band dispersion. It must be stressed that the selection of the layer widths demands more information of the strain impact on the material quality because the SL constituent layers are not lattice matched. In terms of the “Ga-free” InAs/InAsSb T2SLs, a fairly thick InAs layer is needed to balance the strain on the thinner InAsSb (the InAs is under a small tensile strain and InAsSb is under large compressive strain).

In recent years, a new material system based on antimony-strained layer superlattices has emerged, attracting much interest with prons such as high material homogeneity, high bandwidth tunability, and Auger recombination suppression. However, in the case of MWIR APDs based on InAs/InSb T2SLs, their performance is limited by the equality of *α*_*h*_ = *α*_*e*_^71^.

Razeghi et al. have demonstrated the MBE-grown MWIR SAM-APD device [see Fig. 8(f, g)] which consists of AlGaAsSb/InAs_0.9_Sb_0.1_ multi-quantum well as a multiplication layer^73^. The AlAs_0.1_Sb_0.9_/GaSb T2SLs were assumed to be the barrier of the multi-quantum well structure. This design of the multiplication layer provides high flexibility in the energy band engineering, allowing for large differences in electrons and holes ionization rates, which can be seen in Fig. 8(h). The maximum multiplication gain increases from 29 (under −14.7 V) at 200 K to 121 at 150 K.

## Low-dimensional solid avalanche photodetectors

The extraordinary and unusual electronic and optical capabilities of low-dimensional solid materials make them be capable of avalanche photodetector applications. In the last decade, many avalanche photodetectors have been demonstrated using nanowires (coupled with plasmonic and photonic crystals) and two-dimensional (2D) layered materials^8^. So far, however, the main research activity is focused on devices operating in VIS and SWIR regions. For this reason, this section will only briefly describe the most interesting and published results.

The nanoscale photodetectors exhibit relatively low sensitivity. A way to enhance their responsivity is the avalanche multiplication mechanism observed, for example, in Si-CdS *p*–*n* heterojunction photodetector based on nanowire structure, or in an InAsP quantum dot after tunneling into InP avalanche nanowire photodiode^75,76^. In 2019, Farrell et al. published on the isolated absorption and impact ionization regions avalanche photodiode array of 4400 InGaAs/GaAs nanowires^77^. This array design greatly improves the volume of the multiplication area and the number of filled traps. However, this innovative APD design requires a cryogenic operation which limits its widespread applicability.

2D materials originate directly from layered van der Waals (vdW) solids. The plane atoms are coupled by ionic or covalent bonds, while layers are linked by weak vdW interactions allowing that 2D material could be fabricated by mechanical exfoliation from bulk source materials. In addition, weak vdW bonds allow possible combinations of the 2D materials providing flexibility in heterostructure design.

Different types of 2D photodetectors with the flexibility in forming heterostructures have already been widely studied with the advantages of weak vdW interactions. The most popular are photoconductive, photovoltaic, phototransistor (hybrid detectors), and photothermoelectric^78^. However, the avalanche mechanism through impact multiplication has not yet been researched thoroughly in 2D photodetectors. In this review, our discussion is focused on the avalanche effect in 2D layered materials and their vdW heterostructures. 2D layered graphene, being gapless, makes it difficult to construct high detectivity photodetector. On the other hand, an alternative to graphene—2D materials [like black phosphorus (BP), InSe] and their heterojunctions (like BP/InSe, BP/MoS_2_, MoS_2_ (*E*_*g*_ = 1.8 eV)/p-type Si (*E*_*g*_ = 1.1 eV)) exhibit promising avalanche performance in VIS to NIR ranges^8^.

**Fig. 9:**
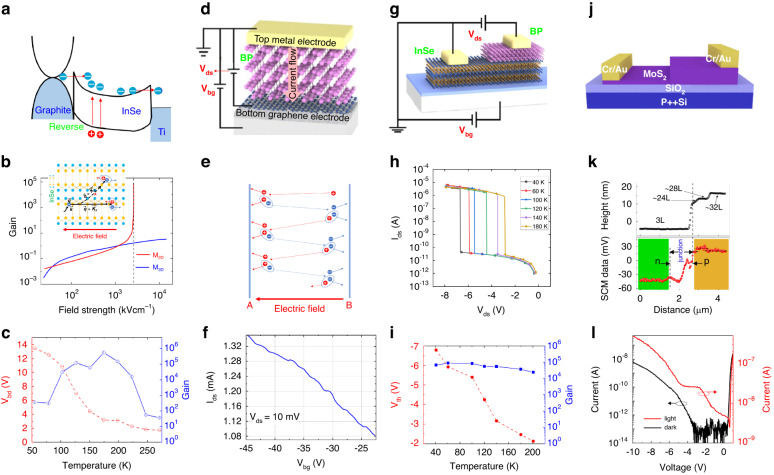
Low-dimensional solid avalanche photodetectors. **a** graphite/InSe Schottky avalanche detector - injection, ionization, collection electron transport mechanisms, **b** e-ph scattering dimensionality reduction affects electron acceleration process and gain versus electric field in 2D (red line) and 3D (blue line), **c** breakdown voltage (*V*_*bd*_) and gain as a function of temperature—exhibits a negative temperature coefficient^81^. Nanoscale vertical InSe/BP heterostructures ballistic avalanche photodetector: **d** schematic of the graphene/BP/metal avalanche device^83^, **e** ballistic avalanche photodetector operating principle, **f** quasi-periodic current oscillations, **g** schematic of the graphene InSe/BP^83^, **h** *I*_*ds*_*–V*_*ds*_ characteristics for selected temperatures (40 − 180 K), **i** avalanche breakdown threshold voltage (*V*_th_) and gain versus temperature—showing a negative temperature coefficient. Pristine PN junction avalanche photodetector: **j** device structure, **k** as the number of layers increases, a positive/negative signal of SCM denotes hole/electron carries, **l** APD’s low temperature (~100 K) dark and photocurrent *I–V* curves^87^

In order to observe the avalanche mechanism, Lei et al. applied more than 50 V reverse bias voltage into a 2D InSe field effect transistor, resulting in a large Schottky barrier between Al/InSe junction on Si substrate and 285 nm-thick SiO_2_ layer^79^. At a bias voltage above 12 V, the *E* in InSe is large enough to speed up photogenerated electrons and generate electron-hole pairs by carrier multiplication. Further increase of the voltage (>50 V) causes the metal/semiconductor junction breakdowns leading to the dramatic rise of both photocurrent and dark current and lowering signal-to-noise ratio. Also, Atalla et al. have observed increasing in photocurrent versus bias voltage in the Ti/BP Schottky barrier due to the avalanche effect^80^. Comparable results were reported by Gao et al. on the avalanche effect in the graphite/InSe Schottky detector [see Fig. 9(a)]^81^. Due to the quantum confinement effect caused by the vdW gap in the layered InSe, two different carrier processes can be distinguished in that device. As presented in Fig. 9(b), the vdW ~1.85 eV gap acts as a tunneling barrier that limits the out-of-plane charge transport, causing the dimensionality of the electron-phonon (e-ph) scattering to decrease and the increase of the Coulomb interaction. As the e-ph scattering is limited the multiplication rate will be boosted resulting in higher *M* at lower breakdown biases. The high gain is reached by the dimensionality reduction of the e-ph scattering in the 2D material which was presented in Fig. 9(b). Unlike conventional avalanche devices holding the positive temperature coefficient of the threshold voltage, the demonstrated device exhibits the negative temperature coefficient presented in Fig. 9(c).

In the case of vertically stacked BP/InSe heterostructure, the ballistic avalanche effect was observed where the carriers ionization probabilities are comparably caused by their symmetric band structure^82^. Zhang et al. also fabricated an InSe/BP heterojunction where the ballistic avalanche effect can be observed^83^. The schematic diagram of the mechanism corresponding to the ballistic avalanche process is presented in Fig. 9(e). The electric field can make the hole accelerate to get enough energy to produce the carriers pair in one pass to plane “A”, and in this way, two holes can be collected, while the electron is transported into the channel. A step further, the electron can generate another electron-hole pair by impact multiplication, and this is collected by plane “B” and the hole drift back to the channel in the repeating cycle.

When the channel length is shorter in comparison to the carrier mean free path, the character of the carrier transfer will change dramatically. Specifically, the transport of electrons within the average free range will no longer be affected by any scattering. That allows to limit noise and power consumption of the photodetector. In Fig. 9(f), the current curves exhibit a quasi-period oscillation denoting the ballistic transport of the BP channel. The InSe/BP device exhibits a negative temperature coefficient, as presented in Fig. 9(i). That comes from the broadening of the Fermi-level and band-bending shift caused by thermal-expansion^83^.

The 2D/3D systems are especially promising for avalanche photodiode technology, where 2D materials can be used for active layers while 3D Si as a multiplication region [see Fig. 9(j)]^84^. Once illuminated, the incident photons generate the carrier pairs being accelerated by bias at the heterointerface. The 2D/3D vdW interface prevents lattice mismatch problems allowing to reach high-quality heterojunctions. As mentioned 2D MoS_2_ proved to be a proper material for APDs fabrication^84–86^. In addition, the APDs can be fabricated by the different number of MoS_2_ layers. Xia et al. reported on the homojunction transistor based on MoS_2_. As the number of layers changes, 2D MoS_2_ exhibits different doping characteristics, as shown in Fig. 9(k)^87^. This natural *p*–*n* homojunction exhibit a well-defined interface. The device under the illumination of 0.42 mW/mm^2^ and wavelength 520 nm with the bias voltage −4.5 V can photogenerate large amounts of electron-hole pairs, as illustrated in Fig. 9(l). For a conventional avalanche conditions, the external electric field is large enough, and electrons or holes can get sufficient energy to achieve avalanche breakdown. It is suggested that the effect is conditioned by the ionization of electrons in the outer layer producing secondary carriers^87^.

The detailed comparison of the APDs performance among 2D material family detectors was presented in Table 7. The responsivity (*R*), response time (*RT*), operating wavelength (*λ*), dark current (*I*_Dark_), external quantum efficiency (*EQE*), normalized photocurrent-to-dark current ratio (*NPDR*), avalanche gain (*M*), and operating temperature (*T*) with proper reference were presented. The highest gain 10000 was reported for BP/InSe (operating wavelength 4 μm, at 10–180 K) and 903 for MoS_2_ (operating wavelength 633 nm, at 300 K)^81,84^.

**Table 7:** Performance comparison of selected 2D photodetectors to include APDs

Device/structure/material	*R* (A/W)	*RT* (ms)	*λ* (nm)	*I*_Dark_ (A)	*EQE*	*NPDR* (W^−1^)	*M*	*T* (K)	Ref.
InSe APD	---	0.06	400–800	1.3 × 10^−9^	3.4	---	152	---	^79^
BP/InSe APD	80	---	4000	---	24.8	---	10^4^–10^5^	10–180	^81^
BP APD	130	---	500–1100	2 × 10^−6^	310	6.5 × 10^7^	7	300	^82^
MoS_2_ APD	2.2	---	633	2 × 10^−7^	---	1.1 × 10^7^	903	300	^84^
Gr–WS_2_–Gr	0.1	---	633	10^–7^	0.3	1 × 10^6^	---	---	^105^
Gr–InSe–Gr	60	0.12	400–1000	5 × 10^−10^	148.5	1.2 × 10^11^	---	---	^106^
Gr–MoTe_2_–Gr	5	0.03	600–1350	---	0.4	---	---	300	^107^
Gr–WSe_2_–Gr	0.04	5.5 × 10^−9^	---	---	0.07	---	---	---	^108^
Gr–WS_2_–Gr	3.5	>2000	532	10^–8^	9.3	3.5 × 10^8^	---	---	^109^
Gr–WSe_2_/GeSe–Gr	6.2	0.03	520	---	14.9	---	---	300	^110^
Gr–WS_2_/MoS_2_–Gr	2.340	>10,000	---	10^–6^	---	2.34 × 10^9^	---	---	^111^
Gr–MoTe_2_–Gr	0.11	0.024	1064	5 × 10^−7^	0.13	2.2 × 10^5^	---	---	^112^
Gr–MoTe_2_–Gr	0.03	6.15 × 10^−3^	550	6 × 10^−8^	---	4.6 × 10^5^	---	300	^113^
BP APD	---	---	532	1.05 × 10^−5^	2.7	---	272	300	^80^
InSe APD	11000	1	405–785	5 × 10^−9^	---	2.5 × 10^12^	500	---	^114^
MoTe_2_–WS_2_–MoTe_2_ APD	6.02	475	400–700	9.3 × 10^−11^	14.1	6.47 × 10^10^	587	295	^115^
APD120A APD	25	---	400–1000	---	---	---	50	---	^115^
LSSAPD9-230 APD	0.57	3 × 10^−5^	400–1000	10^–9^	---	---	60	---	^115^
AD100-8 TO APD	50	1.8 × 10^−5^	400–1100	10^–10^	---	---	100	---	^115^
MTAPD-06-001 APD	50	3 × 10^−5^	400–1100	4 × 10^−10^	---	---	100	---	^115^
InSe	0.244	23	685	---	0.44	---	---	295	^116^
BP	0.0048	1	640	---	0.0093	---	---	295	^117^
MoS_2_	0.0075	50	450–800	---	0.017	---	---	295	^118^
BP/MoS_2_	0.9	2.7 × 10^−3^	2500–3500	---	0.35	---	---	300	^119^
WSe_2_/MoS_2_	88 μA/W	---	532–1030	---	---	---	1300	300	^120^

Large dark current in, e.g., multilayer 2D-based detectors has been found to be a main problem hampering further progress. In order to limit the dark current, the typical approach is the source-drain-gate detector with the ability to carrier concentration monitoring in the channel. In comparison to the two-, the three-terminal detector makes the structure more complicated and the continuous gate voltage is energy-consuming. An additional common approach is to implement heterojunctions formed by the connected TMDs. That may successfully suppress dark current and improve performance, however, the depositing process of TMDs on the different materials is difficult and inefficient, making that technique hard for large-scale applications. Lately, organic-inorganic hybrid perovskites (OIHP) were reported to exhibit the potential to increase detector performance due to remarkable capabilities (broadband absorption coefficient, direct bandgap). In addition, OHIP could be deposited by the not complicated, low temperature, and low-cost spin-coating techniques. By depositing 2D OIHP on a multilayer MoS_2_ device, the nominal dark current was remarkably reduced by six orders of magnitude^88^.

Lately, 2D materials have been implemented to fabricate THz detectors^89^. The bP exhibiting direct bandgap for bulk (*E*_*g*_ ≈ 0.35 eV) and monolayer (*E*_*g*_ ≈ 2 eV) phases, significantly large mobility (>1000 cm^2^ /Vs) make that material an appropriate candidate for the THz detection. Viti et al. presented the bP THz detector operating at 300 K in 2015^90^. Authors used the mechanically SiO_2_-encapsulated bP flake in an antenna-coupled top-gate FET where a typical bonding tape method was implemented to move the flake on a 300-nm SiO_2_ layer on the top of a 300-µm thick Si. The photodetection mechanism in bP-based THz FETs was found to be based on the result of three effects including photothermoelectric, bolometric, and plasma-wave rectification effects^90^. Noise equivalent power (*NEP*) for listed mechanisms reaches ~7 nW/Hz^1/2^, ~10 nW/Hz^1/2^, and ~45 nW/Hz^1/2^ for the bolometer, plasma-wave, and thermoelectric detector, respectively. The responsivity of ~5–8 A/W at 0.3 THz allows to apply a bP FET detector for real-time quality control and pharmaceutical purposes^91^. To avoid influence of the ambient temperature on the exfoliated bP flake, Viti et al. incorporated a bP flake within a multilayered structure to form hBN/bP/hBN THz FET devices allowing to reach *NEP* ~ 100 pW/Hz^1/2^ and voltage responsivity, *R*_*v*_ ~ 38 V/W at 4 K (at 295 GHz) and ~10 nW/Hz^1/2^ and ~2 V/W at 300 K, respectively. Viti et al. presented the latest progress on the bP photodetectors operating in the spectral range 0.26–3.4 THz focusing on the possible issues and challenges in the device’s processing and fabrication^91,92^.

Lately, it has been presented that topological insulators (TI) exhibit potential for a wide spectral range including THz detection. TIs are being considered as an advanced quantum phase of matter, characterized by a semiconducting bulk and topologically protected surface states with a spin and momentum helical locking and the Dirac-like band structure^93,94^. 2D TIs could be connected with gapless edge states and 3D insulators with gapless topological surface states (TSS)^95^.

An advantage of THz plasmonic with TIs is connected with the THz radiation rectification via excitation of plasma waves in the antenna-coupled FETs active channel. The very first presentation of THz detection facilitated by TSS in top-gated nanometer FETs using thin Bi_2_Te_3−*x*_Se_*x*_ flakes was shown by Viti et al. in ref. ^96^. The maximum *R*_*v*_ ~ 3.0 V/W and the minimum *NEP* ~ 10 nW/Hz^1/2^ was reached for 292.7 GHz. Yao et al. presented TI THz heterojunction Bi_2_Te_3_-Si device^97^. The pioneering approach for THz detection at 300 K using a subwavelength metal-Bi_2_Se_3_-metal structure exhibiting 300 K *R*_*i*_ ~ 75 and 475 A/W for 0.3 THz operating in the self-powered and voltage modes was shown by Tang et al.^98^. The measured *NEP* ~ 3.6 × 10^−13^ W/Hz^1/2^ and D^*^ ~ 2.17 × 10^11^ cmHz^1/2^/W were reached for *V* = 50 mV.

## Avalanche photodiodes in active imaging systems

Thermal imaging systems are divided into passive and active devices. The typical night vision system is based on thermal imaging cameras. In this case, the imaging device does not emit any energy but only acts as a receiver. Conversely, when a source is used to light and gather the reflection from the target, the camera can be considered an active system allowing to obtain images during the day and night, under different illumination conditions.

**Fig. 10:**
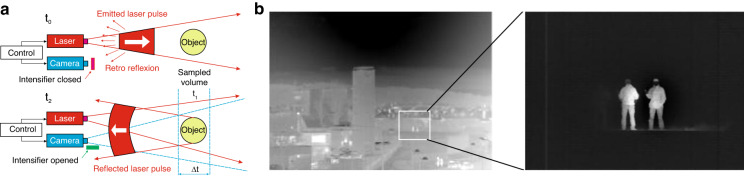
An idea of laser-gated system connected with passive thermal imaging for enhanced distant identification. **a** operation principle [at *t*_0_—camera is closed—light pulse is emitted, at *t*_1_—target reflects light pulse, at *t*_2_—the camera is opened for a short period (*∆t*) matching the needed depth of view]; **b** typical images of wide *FOV* thermal and laser-gating systems^53^

Figure 10(a) illustrates the range-gating technology system in conjunction with other sensors. The range-gating technology consists of a pulse laser (typical wavelength, *λ* = 1.55 μm), laser receiver (for ranging), gated detector, wide field of view (*FOV*) thermal imager and monitor electronics. A light pulse is emitted toward an object. Once the reflected light returns from the target, the accompanied high-speed electronic shutter activates at the appropriate moment. The detector must meet stringent requirements for high sensitivity and extremely high-frequency response and is a main, performance-driven part of the system.

The gating technology allows to select an exact piece of space so that operator can see the target location, without parasitic lights or light scattering by aerosol particles. Selecting gating width (a narrow enough slice of space), the system significantly increases the detectivity. The typical images generated by the wide *FOV* thermal camera and the laser-gated imaging are shown in Fig. 10(b).

Between different active imaging systems, 3D pulsed-laser LIDAR using APD arrays has drawn attention due to its simple operation principle, high interference immunity, and long imaging distance range^99,100^. There are two types of flash LIDARs available: the linear and the Geiger modes. When linear mode is activated, the reverse voltage on the APD is lower than the breakdown bias and carriers are taken up faster than being generated, causing the avalanche process to terminate itself. In this case, the output photocurrent generated by the finite gain is a linear function of the echo pulse intensity. The APD operates at Geiger mode when the reverse voltage exceeds the APD’s breakdown bias. Photoexcitation of the single carrier can cause the multiplication current peak to be high enough to be detected by the threshold detection circuit, making the detection process noiseless because it is inherently digital.

The APD’s spectral response depends on the material used in the absorption region. Silicon APDs have a sharp cut-off wavelength close to 1 μm. In this case, a 905 nm light pulse can be absorbed in eye vitreous humor and lead to possible retina destruction at fairly moderate laser powers (the laser is focused by the lens on the small retina spot). The systems equipped with InGaAs APDs can use lasers being safe for the eye (traditionally 1.55 μm) to minimize damage to the users’ eye (the SWIR beam with a wavelength exceeding ~1400 nm is powerfully absorbed by eye parts before reaching the retina). HgCdTe APDs operate in linear mode and exhibit *QE* close to 90% in a wavelength range of 5 μm. Table 8 collects the pulsed-laser 3D imaging flash LIDAR performance incorporating the linear-mode APD arrays.

**Table 8:** Parameters of pulse-laser 3D imaging flash LIDAR by APDs arrays

Company/Institution	Energy per pulse	Wavelength	Pulse width	Repetition frequency	Array scale	Material	Field of view	Distance	Accuracy	Frame rate	Ref.
Advanced Scientific Concepts	---	1570 nm	5 ns	---	128 × 128 (PIN)	InGaAs	---	0.06 m–1.1 km	15 cm	1–20 Hz	^121^
Advanced Scientific Concepts	---	1570 nm	5 ns	---	128 × 128 (PIN)	InGaAs	---	2 km	0.1 m	10–60 Hz	^122^
Advanced Scientific Concepts	---	1570 nm	5 ns	---	32 × 32	InGaAs	---	---	---	---	^123^
Advanced Scientific Concepts	11 mJ	1064 nm	---	---	128 × 128	InGaAs	---	---	25 cm@200 m	---	^124^
Ball Aerospace and Technologies Corporation	---	1570 nm	5 ns	---	128 × 128	InGaAs	---	1.8 km	---	30 Hz	^125^
Raytheon	---	1500 nm	---	---	256 × 256	HgCdTe	6^o^ ~ 24^o^	7–10 km	5 cm@1 km	30 Hz	^126,127^
DRS Technologies	30 mJ	1570 nm	8 ns	30 Hz	128 × 128	HgCdTe	---	2–10 km	---	---	^102^
CEA/LETI	8 mL	1570 nm	8 ns	---	320 × 256	HgCdTe	---	40 m	11 cm	7–74 Hz	^128^
Guilin University of Technology	8.8 mJ	905 nm	8 ns	25 Hz	5 × 5	Si	1.2^o^ ~ 1.2^o^	15 m	11 cm@15 m	---	^129^
Harbin Institute of Technology	5–30 μJ	532 nm	10 ns	5–15 Hz	8 × 8	Si	---	5 m	~0.4 m	---	^130^
South-West Institute of Technical Physics of China	100 mJ	1064 nm	5 ns	20 Hz	64 × 64	InGaAs	---	1 km	0.15 m	---	^131^

The putting the new APDs technology into the market, except for performance and potential applications, two key factors should be taken into consideration: fabrication readiness and budget efficiency. Currently, a uniform integration process must be developed to allow the 2D material-based APDs to be matching with the current CMOS technology to reach improved parameters at a reasonable cost. Table 9 compares the existing and well-developed A^III^B^V^, A^II^B^VI^ material technologies with emerging 2D materials for APDs fabrication^101^. It must be underlined that even though both A^III^B^V^ and A^II^B^VI^ materials have established themselves as standard for APDs and hold the leading position in the existing IR market, certain elementary restrictions which have not been circumvented yet. It must be underlined that after 60 years of technological development, the ultimate A^III^B^V^ and A^II^B^VI^ APDs HOT detection parameters have not been reached. The A^II^B^VI^ (HgCdTe) semiconductor instability and high lattice mismatch (A^III^B^V^ materials) generated strain create defects limiting the devices’ performance. Another key issue is the high fabrication/processing cost, extremely complicated growth techniques, and sophisticated device architectures. 2D APDs on the other hand, could be easily processed to include device design, substrates selection, and fabrication methods. The 300 K operation of 2D material-based APDs is the most crucial feature conditioning their cost-effectiveness. The 2D material-based APDs have been reported to exhibit the remarkable capability to substitute the typical APDs in relation to the gain, dark current suppression, excess noise, *I*^*2*^*E* engineering, and operating temperature. It must be stressed that the thin layers building 2D APDs enable flexibility in impact ionization coefficients tuning leading to dark current suppression and low power usage in comparison to commercial devices based on well-developed bulk materials.

**Table 9:** Comparison of A^III^B^V^, A^II^B^VI^ (HgCdTe), and 2D-based APDs technologies

Material	Advantages	Disadvantages
A^III^B^V^	Monolithic integration is possible. Sophisticated/Developed technology. Short-time response operation.	Low operating temperature to suppress thermal noise and enhance D^*^. Large lattice mismatch -heteroepitaxial deposition—influencing device performance. High fabrication cost.
A^II^B^VI^	Multiband operation/detection—flexibility in the bandgap tailoring. High absorption coefficients leading to high *QE*.	Weak Hg-Te bonds causing bulk, surface, interface instability. Non-uniformity—large area growth. High fabrication cost. Difficult/sophisticated growth techniques and device structure.
2D	300 K operating temperature. Thickness-dependent material properties. *I*^*2*^*E* device structures—possible integration with 0, 1, 2, 3 D materials. Cost-effective.	Inherently low light absorption and short carrier lifetime. Lack of large-scale deposition methods. Passivation is needed to protect the surface.

## Conclusion

The detectors for optical telecommunication applications and quantum information technologies have mainly pushed the APDs to progress with high *BW*, low *F(M),* and high *GBW* from 1975. It was shown that the APD provides better parameters in comparison to typical p–n or p-i-n-based devices including detectivity, gain, and time response. It is visible that APDs have been successfully applied into the variability of applications, however, the chase to suppress the random noise [to achieve *F(M)* < 2] related to the multiplication nature has been constant because the excess noise restricts the detector’s sensitivity, detectivity and reduces the operating *BW*. The solution is a higher and fully controlled—deterministic impact ionization mechanism which can be achieved either by the proper multiplication material selection, by device design (scaling/thin multiplication regions), or by material engineering. It was demonstrated that the non-local effect of impact multiplication allows to limit of the noise in many materials covering the wide radiation range. In addition, the “*third wave*” materials and related technologies have opened the prospect of *I*_*2*_*E* to design and fabricate heterojunctions to further suppress noise and reach higher *GBW*.

It must be stressed that the APD’s yield is highly conditioned by sufficient *GBW* being strictly related to the *F(M)*. The conditions and variables allowing to reduce noise contribute also to high *GBW*. Therefore, the *GBW* increase and *F(M)* suppression have been an effort for the progress and investigation. The following methods to improve APDs performance must be implemented:

- 1) choosing a material with advantageous carrier multiplication coefficients. The APD’s avalanche layer contributes to the *M*, *F(M)*, and *GBW* products. The local-field multiplication model explains that the APDs *F(M)* and *GBW* are conditioned by the material’s carriers multiplication coefficients in the avalanche layer. Higher detection parameters are reached if one of the multiplication coefficients is substantially higher than the other, i.e., the *k* = *α*_*h*_*/α*_*e*_ differs significantly from unity. Attempts to increase APD’s parameters have moved to electric field profile optimization and research on the new compounds to include bulk A^III^B^V^, A^II^B^VI^, “*third wave*” materials and technologies—T2SLs InAs/GaSb, “Ga free”—InAs/InAsSb and 2D materials. Flexibility in bandgap energy tuning and the energy band profile optimization in 2D materials makes the impact ionization be monitored by the number of layers modification. It is feasible to adjust the *k* = *α*_*h*_*/α*_*e*_ level by varying the number of 2D materials constituting layers. For bulk and “*third wave*” avalanche layers, the minimal *F(M)* has been reached with materials such as Si, HgCdTe, InAs, Al_*x*_Ga_1–*x*_As_*y*_Sb_1–*y*_, Al_*x*_In_1–*x*_As_*y*_Sb_1–*y*_, T2SLs InAs/GaSb, and MoS_2_ exhibiting *k* « 1);
- 2) thickness reduction (scaling) of the avalanche layer to utilize the non-local nature of the multiplication process [reducing the thickness of the multiplication layer leads to lower *F(M)*]—proved for many bulk materials used for avalanche regions: InP, GaAs, In_1–*x*_Al_*x*_As, Si, Al_*x*_Ga_1–*x*_As, SiC, GaP, GaInP). As 2D materials-based APDs are inherently reduced to the submicrometer level, the absorbers based on those materials are under large lateral electric fields leading to the breakdown;
- 3) *I*_*2*_*E* using appropriately designed heterojunctions. The lowest *F(M)* could be reached by using impact ionization layers where electrons are transported from a wide energy bandgap material to the bordering low bandgap semiconductor. The electrons’ energy increases in the wide bandgap layer but high threshold energy prevents them from multiplication. Next, the high energy electrons are transported to the low threshold energy, narrow bandgap layer where they are being immediately multiplicated. The conduction band discontinuity ensures extra energy to enhance that process. The generated holes are promptly transported to the wide bandgap layer where multiplication is much more limited. It must be stressed that both effects reduce *F(M)* due to the fact that gain is much more one-carrier prompted and occurs with a higher probability. Among the most commonly used heterojunctions could be listed: (GaAs/Al_*x*_Ga_1–*x*_As, In_0.52_Al_0.48_As/In_0.53_Ga_0.17_Al_0.3_As, InAlAs/InAlGaAs—cascade/tandem/multistage structures, Al_*x*_Ga_1–*x*_As/GaAs and Al_0.7_In_0.3_As_0.31_Sb_0.69_/InAs_0.91_Sb_0.09_—staircase, InSe, BP/InSe, MoS_2_, BP, MoTe_2_–WS_2_–MoTe_2_, 2D vdW). Here, 2D material-based APDs exhibit potential in developing ultrathin and favorable miniature devices. Typical bulk materials APDs are restricted by reasonably high dark currents. That problem could be resolved by nanomaterials and nanostructures incorporation (due to the Schottky barriers) as APDs absorbers.

The APDs can operate below or above breakdown bias for many applications. When the APD operates below breakdown voltage, the avalanche gain is fixed, meaning that the device may be used for photon energy selection, while when the detector operates above the breakdown bias (Geiger mode: single-photon detection regime), the photon may activate multiplication breakdown, causing substantial carrier avalanche allowing single-photon detection. Recently an impressive increase in interest in new SPD technologies has been observed due to massive internal gain, short-time response, high sensitivity, small volume, and flexibility in integration. Its device performance including SPADs has been increased via external quenching circuits and device structure optimization. The main reason for that trend is unquestionably the move for QKD. Effective single-photon counting, with a single-photon detection efficiency >50% was reached only for wavelengths <2 μm. That spectral region is mainly covered by SNSPDs providing remarkable performance but their applications are restricted by the cryogenic cooling requirements. Conversely, SPADs circumvent the inherent restrictions of SNSPDs by possible 300 K operation by A^III^B^V^ material leader—InGaAs. Extension of the SPD performance to MWIR (>2 μm) exhibits prospective to be applied in astronomy, LIDAR, research on dark matter, and the elementary investigation of molecules.

Once APDs based on typical bulk materials have reached a high level of development and are broadly used for quantum information purposes for single-photon detection, to meet the demanding technologies in the long-range field such as FSO, LIDAR/LADAR, ToF, intelligent robotic and in battlefield conditions (military applications) the 2D material detectors are speedily designed, developed, assessed and implemented. 2D semiconductors allow implementing of new approaches for sophisticated APDs’ development by effective carrier ionization at the low-dimensional level enabling broad potential in the area of photon-counting purposes.

Further optimization of the APD performance is possible allowing to design and fabrication of devices with supreme parameters over conventional avalanche devices. For instance, by choosing 2D materials with promising band alignments and structures, it is feasible to implement appropriate Schottky junctions to suppress the dark currents and widen operating wavelengths. Moreover, improvement in processing allows us to reduce the response time and current noise. The 2D APD has been reported to be operating within VIS, NIR and MWIR ranges with a *R*_*i*_ ~ 80 A/W, *EQE* ~ 24.8%, and *M* ~ 10^5^ for MWIR [*λ* = 4 μm, *T* = 10–180 K, BP/InSe APD].

That paper has reviewed the multiplication effect generated by the avalanche process and sketchily reviewed the latest research on bulk and “*third wave*” APDs. The progress in the development of the APD operating in the IR range was presented covering materials based on HgCdTe as well as A^III^B^V^ alloys including “Ga-based” and “Ga-free” T2SLs. The non-local characteristic approach and technological achievements have opened up the option of multiplication engineering incorporating different materials and heterojunctions to reach better performance: suppressed noise with higher *GBW* in broader spectral regions. It is believed that the 2D/vdW APD could prove itself to be an alternative to the bulk multiplication devices providing a possible method for developing devices exhibiting high sensitivity and low excess noise.

## Data Availability

All data generated or analyzed during this study are included in this published article.
